# Disease of the Tibia from Injury

**Published:** 1844-06-29

**Authors:** 


### DISEASE OF THE TIBIA FROM INJURY.

Dr. Chisholm related in the London and Edinburgh Medical Journal, the case of a man forty years of age, who was admitted into the Northern Infirmary, Inverness, with disease of the left tibia, following a blow from a stone inflicted a twelvemonth previously. On examination, a large ulcerated excavation, about four inches by three, was observed occupying part of the middle and inferior thirds of the leg on its inner and anterior aspect. No bone could be felt in the cavity, and the probe passed down to below the external malleolus. Several fistulous communications were also found on the inner side of the limb around the ankle joint. Numerous pieces of bone had been discharged. In consultation on the case, amputation was unanimously resolved on, but previously to its performance, the anterior tibial artery became involved in the ulceration, and hæmorrhage to a very large extent was the result. The operation was consequently performed as speedily as possible, and the man ultimately recovered without having had a bad symptom.

